# Susceptibility to obesity and gallbladder stasis produced by a protein- and fat-enriched diet in male mice compared with female mice

**DOI:** 10.1186/1743-7075-4-14

**Published:** 2007-06-05

**Authors:** Kyoko Miyasaka, Setsuko Kanai, Minoru Ohta, Ayako Sekime, Saeko Akimoto, Soichi Takiguchi, Akihiro Funakoshi

**Affiliations:** 1 Department of Clinical Physiology, Tokyo Metropolitan Institute of Gerontology, 35-2 Sakaecho Itabashiku 173-0015, Tokyo, Japan; 2 Department of Clinical Research, National Kyushu Cancer Center, 3-1-1 Notame, Minamiku Fukuoka 811-1396, Japan; 3 Division of Gastroenterology, National Kyushu Cancer Center, 3-1-1 Notame, Minamiku Fukuoka 811-1396, Japan

## Abstract

**Background:**

The frequency of Japanese subjects over 20 years old with metabolic syndrome is 45.6% in men but just 16.7% in women. The reason why Japanese male subjects are more susceptible to metabolic syndrome than women is unknown. One possibility is the higher frequency of Japanese male subjects (40–70 years old) who had a drinking habit (67%), while that of female subjects was only 25%. In addition, daily fat intake was markedly increased in Japanese subjects (from 9% to 25%), and cholesterol cholelithiasis is one of the most rapidly increasing digestive diseases during the past 50 years. The object of this study is to examine whether a potential sex-related risk factor exists in the manifestation of metabolic syndrome as well as gallstone formation.

**Methods:**

Gallbladder dysmotility accerelates gallstone formation and gallbladder contraction depends on cholecystokinin (CCK) and its receptor (CCK-1R). We developed CCK-1R gene knockout (-/-) mice. The effects of the fat- and protein- enriched diet OA-2 on body weight, hyperlipidemia, and frequencies of sludge and gallstone formation were examined, and compared between wild-type and CCK-1R(-/-) male and female mice. The OA-2 diet contains slightly higher protein and fat (7.9 % fat and 27.6 % protein) compared with a standard diet (CRF-1) (5.6 % fat and 22.6 % protein), but their total energies are similar. After weaning, CRF-1 was provided until 3 months of age in all animals. Administration of an OA-2 diet was started when age-matched CCK-1R(-/-) and wild-type male and female mice reached maturity, at 3 months of age. Administration of CRF-1 was continued in the rest of the animals. Mice were sacrificed by guillotine at 6 and 12 months of age and the blood was collected to measure plasma levels of triglyceride and cholesterol. The gallbladder was removed and classified as normal (clear gallbladder), clouded (sludge formation), and/or containing gallstone formations.

**Results:**

As long as CRF-1 was provided, the frequency of sludge and/or gallstone formation in CCK-1R(-/-) male mice was 3 of 8 (35%) and 4 of 9 (45%) in females at 12 months of age, whereas no gallstone formation was observed at 6 months of age. On the other hand, male mice fed OA-2 increased their body weight and plasma lipid concentrations, compared with those fed CRF-1 regardless of genotype. Under the OA-2 diet, sludge and gallstone formation was observed at 6 months of age, not only in CCK-1R(-/-) male mice but also in wild-type male mice. In contrast, parameters in female mice did not differ between the two diets.

**Conclusion:**

Male mice were more susceptible to protein- and fat-enriched diet-induced obesity than female mice, and hyper-nutritional status accelerated sludge and gallstone formation in male mice.

## Background

According to the recent report by the Ministry of Health, Labour and Welfare of Japan [1], the percentages of protein, fat, and carbohydrate of daily food intake in 1957 were 13%, 9%, and 78%, respectively; in turn, in 2003, these percentages became 15%, 25%, and 60%. Thus, daily fat intake was markedly increased in Japanese subjects. The frequency of Japanese subjects over 20 years old with obesity (as measured by waist circumference) plus either one or two factors (plasma HDL cholesterol level < 40 mg/dl, systolic blood pressure > 130 mmHg and diastolic blood pressure > 85 mmHg, and/or HbA1c > 5.5%)(so called metabolic syndrome) is 45.6% in men but just 16.7% in women. In contrast, the percentage of daily energy intake from dietary fat is not higher in men than in women. The reason why Japanese male subjects are more susceptible to diet-induced obesity than women is unknown. One possibility is the higher frequency of Japanese male subjects (40–70 years old) who had a drinking habit (67%), while that of female subjects was only 25% [2]. There have been reports [3,4] that alcohol ingestion increased meal size and energy intake.

In addition, epidemiologic data show [5] that cholesterol cholelithiasis is one of the most rapidly increasing digestive diseases during the past 50 years because of changing lifestyles and nutritional status; namely, a higher intake of fat and protein among the Japanese people. It has been accepted that obesity and hyperlipidemia as well as insulin resistance and hyperglycemia are risk factors for gallstones [6,7]. The formation of cholesterol gallstones requires 3 pathogenic factors: supersaturation of bile with cholesterol, gallbladder dysmotility and stasis, and accelerated nucleation of cholesterol crystals in the gallbladder [8-11], although it is not elucidated whether gallbladder dysmotility is a primary factor in cholesterol gallstone diseases or secondary to inflammation and excess cholesterol accumulation in gallbladder smooth muscle [12,13].

We have cloned the genomic structures of cholecystokinin (CCK)-1R [14] in rats [15], mice [16], and humans [17], and generated CCK-1R gene knockout (-/-) mice [18]. As previously reported by Kopin et al. [19], the lack of CCK-1R itself does not seem to modify changes in body weight or induce glucose intolerance [18]. Gallbladder contraction was not induced by CCK in CCK-1R(-/-) mice, while CCK strongly induced gallbladder contraction in wild-type CCK-1R(+/+) mice [20]. We reported in a previous study [21] that the frequency of sludge and/or gallstone formation in CCK-1R(-/-) male mice was 3 of 8 (35%) at 12 months of age and 6 of 17 (37%) at 24 months of age, whereas no gallstone formation was observed in wild-type mice at any of age. Thus, CCK-1R(-/-) mice can be considered a good model for gallstone formation.

In the present study, we examined whether a potential sex-related risk factor exists in the manifestation of metabolic syndrome as well as gallstone formation. We examined the effect of a diet containing slightly higher fat and protein compared with a standard diet on body weight, plasma lipid concentrations and gallstone formation in wild-type and CCK-1R(-/-) male and female mice.

## Methods

The present experimental protocol was reviewed and approved by the appropriate committee of the Tokyo Metropolitan Institute of Gerontology.

### Animals

The progenitor strain for CCK-1R(-/-) was C57B2/6J [18,20]. More than seven generations of backcrossing were completed. Age-matched male and female littermates CCK-1R(-/-) and wild-type were used for experiments. Mice were maintained in individual cages in a temperature-controlled room at 21°C with 55 ± 5% humidity, and with a 12-h light/12-h dark photocycle (08:00–20:00) at the Tokyo Metropolitan Institute of Gerontology.

### Materials and chemicals

A standard diet (CRF-1, Charles River) was purchased from the Oriental Co., Tokyo, and a breeding diet (OA-2) was purchased from Nippon CLEA Co., Tokyo. The OA-2 diet contains slightly higher protein and fat (7.9 % fat and 27.6 % protein) compared with a standard diet (CRF-1) (5.6 % fat and 22.6 % protein), but their total energies are similar (Table 1).

**Table 1:** Compositions of diets (g/100 g)

diets	CRF-1(standard diet)	OA-2
water	8.1	9
**protein**	**22.6**	**27.6**
**fat**	**5.6**	**7.9**
minerals	6.6	7.2
fiber	3.3	4.5
carbohydrates	53.8	43.8

calories (kcal/100 g)	356	356

### Experimental protocols

After weaning, CRF-1 was provided until 3 months of age in all animals. Administration of an OA-2 diet was started when age-matched CCK-1R(-/-) and wild-type male and female mice reached maturity, at 3 months of age. Administration of CRF-1 was continued in the rest of the animals. The frequencies of sludge and gallstone formations were determined when mice were 6 and 12 months old. Before this determination, we estimated daily food intake over 3 days by providing a single weighed (110 – 120 g) serving of food to individually housed mice; after the 3-day period, the remaining food including crumbs was weighed, and the daily food intake was estimated. Mice were sacrificed by guillotine and the blood was collected into tubes containing EDTA to obtain plasma. Thereafter, the gallbladder was removed and classified as normal (clear gallbladder), clouded (sludge formation), and/or containing gallstone formations. The plasma levels of triglyceride and cholesterol were measured.

### Statistical analysis

Values are expressed as means ± SE. The significance of the differences was assessed by one-way or two-way analysis of variance (ANOVA), followed by Fischer's protected least significant difference (PLSD) test. The frequencies of gallstones with respect to genotypes and sex were analyzed using the chi-squared test. A value of p < 0.05 was considered statistically significant.

## Results

### Diet-based differences in male mice

**Figure 1:**
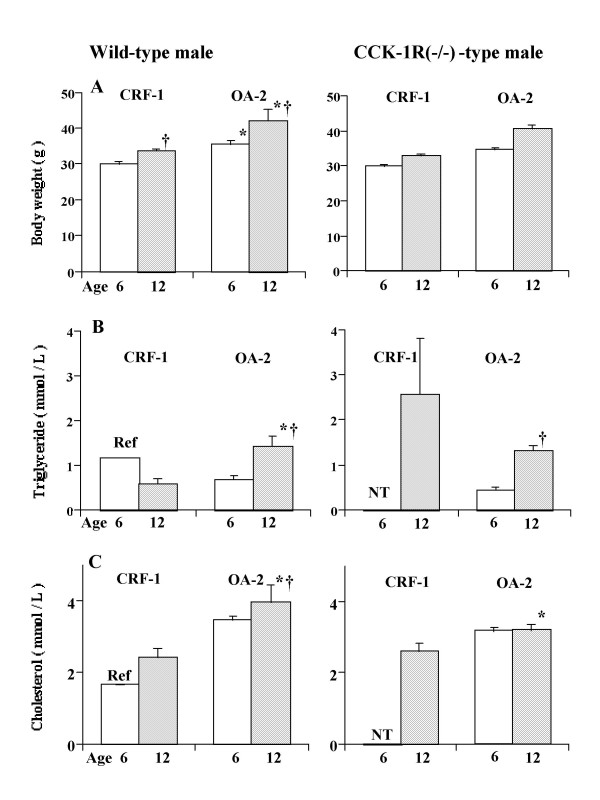
Body weights (A), plasma lipid concentrations (B and C) in wild-type (left panel) and CCK-1R(-/-) male mice (right panel) at 6 and 12 months of age. Body weights and plasma lipid concentrations were higher in mice fed OA-2 than those fed CRF-1 regardless of genotype. *, significantly different from the respective values of CRF-1 mice; †, significantly higher than the respective values at 6 months of age. Values are means ± SE. The results of statistical analyses are shown in the text. The numbers of animals are shown in Table 2. Ref, the value was referred from the record of the animal laboratory of our institute; NT, not tested in the present study.

The body weight was significantly higher in male mice fed OA-2 than in corresponding types fed CRF-1, but it did not differ between CCK-1R(-/-) and wild-type mice (Fig. 1A). Daily food intake did not differ between the two diets. Daily food intake at 6 months of age was as follows: 3 – 4.4 g (mean, 3.89) for wild-type mice fed CRF-1 vs. 2.6 – 4.4 g (mean, 3.69) for CCK-1R(-/-) mice fed CRF-1, and 2.5 – 4.5 g (mean, 3.71) for wild-type mice fed OA-2 vs. 2.82 – 4.43 g (mean, 3.43) for CCK-1R(-/-) mice fed OA-2. Daily food intake at 12 months of age did not differ between the two diets, although the values were lower than those at 6 months of age (mean values are around 3.0 g).

When CRF-1 was provided, no sludge or gallstone formation was observed at 6 months of age regardless of genotype. At 12 months of age, 3 of 8 CCK-1R(-/-) male mice showed sludge and gallstone formation, whereas no sludge or gallstone formation was observed in wild-type mice (χ^2 ^= 8.40, p = 0.004) (Table 2). As neither obesity nor sludge or gallstone formation was observed in CRF-1 fed mice at 6 months of age, plasma lipid concentrations were not examined. The mean values of this age in C57BL mice in the animal laboratory of our institute were 1.15 mmol/L for triglycerides and 1.65 mmol/L for cholesterol, respectively (Fig. 1BC).

**Table 2:** Frequencies of sludge and gallstone formation in 6- and 12-month-old male and female mice

diets	CRF-1		OA-2	
Genotype and sex age	6 months	12 months	6 months	12 months

Male wild-type	0/8	0/20	3/22	2/9*
Male CCK-1R(-/-)	0/20	3/8† #	5/18*	13/18† #
Female wild-type	0/9	1/16	0/8	1/11
Female CCK-1R(-/-)	0/9	4/9†#	1/10	9/15† #

Values are means ± SE. The frequencies of sludge and gallstone formation represent the number of sludge and gallstone formations/the number of animals examined. *, significantly different from the value of the corresponding CRF-1 fed mice; †, significantly higher than the respective values at 6 months of age; and #, significantly different from the value of wild-type mice. The results of statistical analyses are shown in the text.

On the other hand, when OA-2 was provided, sludge and gallstone formation was substantially observed at 6 months of age in both genotypes, though without significant differences between the two genotypes (χ^2 ^= 1.237, p = 0.266)(Table 2). The frequency of sludge and gallstone formation in CCK-1R(-/-) mice fed OA-2 at 6 months of age (5 of 18 mice) was significantly higher than that of those fed CRF-1 (0 of 20 mice)(χ^2 ^= 6.397, p = 0.011), but this frequency in wild-type mice (3 of 22 mice) was not significantly different from the corresponding value (0 of 8 mice) of those fed CRF-1 (χ^2 ^= 1.212, p = 0.271). The frequency of sludge and gallstone formation significantly increased with age in CCK-1R(-/-) mice (χ^2 ^= 7.11, p = 0.008), whereas no age effect was observed in wild-type mice (χ^2 ^= 0.348, p = 0.555). The frequency (13 of 18 mice) in CCK-1R(-/-) mice at 12 months of age was significantly higher than that of wild-type mice (2 of 9 mice) at 12 months of age (χ^2 ^= 6.08, p = 0.014). The difference between CRF-1 and OA-2 in 12- month-old CCK-1R(-/-) mice did not achieve significance (3 of 8 mice for CRF-1 vs. 13 of 18 for OA-2, χ^2 ^= 2.821, p = 0.093); however, the frequency (2 of 9 mouse) of 12-month-old wild-type mice fed OA-2 was significantly higher than that (0 of 20 mice) of mice fed CRF-1 (χ^2 ^= 4.770, p = 0.0029).

The plasma triglyceride concentration increased with age [F(1,63) = 46.8, p < 0.0001] (Fig. 1B), but was not significantly different between the two genotypes (p = 0.153). Aging did not affect the plasma cholesterol concentrations, but all values for OA-2 were higher than the values for CRF-1 (Fig. 1C).

### Diet-based differences in female mice

**Figure 2:**
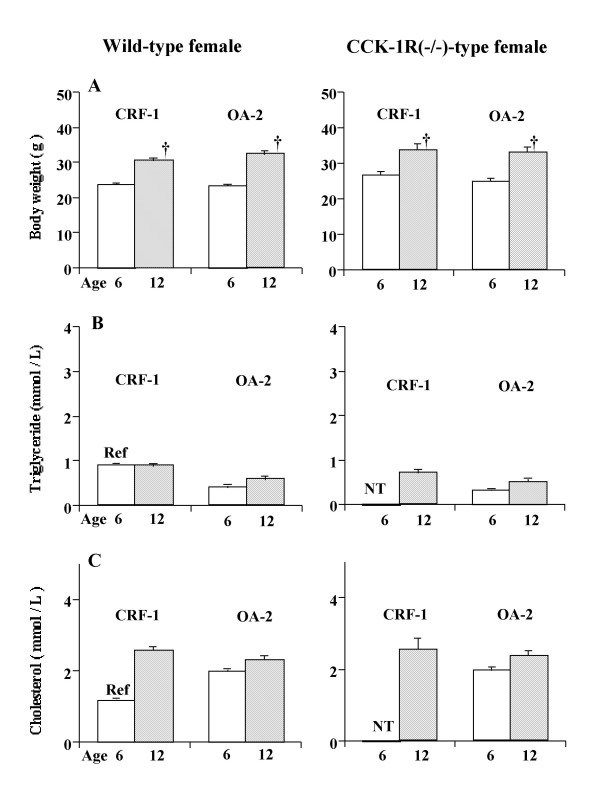
Body weights (A), plasma lipid concentrations (B and C) in wild-type (left panel) and CCK-1R(-/-) female mice (right panel) at 6 and 12 months of age. Parameters are not different with respect to diet or genotype. †, significantly higher than the respective values at 6 months of age. Values are means ± SE. The results of statistical analyses are shown in the text. The numbers of animals are shown in Table 2. Ref, the value was referred from the record of the animal laboratory of our institute; NT, not tested in the present study.

The body weight of 6-month-old female mice fed OA-2 was somewhat lower than that of those fed CRF-1, although the difference was not statistically significant (Fig. 2A). Daily food intake did not differ between the two diets (data not shown).

The distribution of frequencies of sludge and gallstone formation was similar between the two diets (Table 2). At 6 months of age, one CCK-1R(-/-) mouse fed OA-2 showed sludge formation; others showed no sludge or gallstone formation regardless of diet. Aging did not significantly increase the frequencies of sludge or gallstone formation in wild-type mice fed either diet. In contrast, in CCK-1R(-/-) female mice, aging increased the frequency of sludge and gallstone formation significantly (χ^2 ^= 6.258, p = 0.012), and diet was not a significant factor in this: 4 of 9 CCK-1R(-/-) female mice fed CRF-1 and 9 of 15 CCK-1R(-/-) female mice fed OA-2 had sludge and gallstone formation at 12 months of age (χ^2 ^= 0.548, p = 0.459). The frequency in 12-month-old CCK-1R(-/-) female mice was again significantly higher than that in wild-type mice regardless of diet (χ^2 ^= 5.252, p = 0.0219 for CRF-1, χ^2 ^= 6.95, p = 0.008 for OA-2).

In contrast to the results for male mice, the plasma levels of cholesterol and triglycerides in both 6- and 12-month-old females of either genotype did not differ between the two diets (Fig. 2BC).

## Discussion

The effects of administering OA-2 differed between male and female mice. OA-2 contains slightly higher protein and fat compared with CRF-1, but their total energy contents are the same. Daily food intake did not differ between two diets; still, the body weight and plasma lipid concentrations were significantly higher in male mice fed OA-2 than those fed CRF-1, while those values in female mice remained essentially the same. These effects were similarly observed in both wild-type and CCK-1R(-/-) mice.

In spite of no sludge or gallstone formation at 6 months of age in either wild-type or CCK-1R(-/-) male mice fed CRF-1, the frequency of sludge and gallstone formation was substantially observed at 6 months of age in both wild-type and CCK-1R(-/-) male mice fed OA-2 [3 of 22 mice for wild-type and 5 of 18 mice for CCK-1R(-/-)]. Although we examined only 8 mice at 6 months of age in the present study, we conducted a number of experiments using this age of mice [18,20,22] and found no gallstones. The frequency at 12 months of age in wild-type male mice (2 of 9 mice) fed OA-2 was significantly higher than that in those fed CRF-1 (0 of 20 mice). Taken together, these observations suggest that obesity and hyperlipidemia may be responsible for the sludge and gallstone formation wild-type male mice fed OA-2. On the other hand, 12-month-old CCK-1R(-/-) male mice fed OA-2 showed the highest frequency of sludge and gallstone formation (13 of 18 mice, 72%). It could be concluded that such high frequency may be produced by the lack of gallbladder motility due to the lack of CCK-1R plus hyperlipidemia due to obesity, although the difference in frequency between CCK-1R(-/-) mice fed CRF-1 (3 of 8 mice) and those fed OA-2 did not quite show statistical significance (p = 0.093).

In contrast to our findings for male mice, diet did not cause significant differences in the weights of female mice. This result seems rather reasonable, because the energy intake between two diets is the same. Moreover, the frequencies of sludge and gallstone formation in 12-month- CCK-1R(-/-) female mice fed CRF-1 (4 of 9) did not differ significantly from that of the corresponding female mice fed OA-2 (9 of 15).

The reason why the effects of an OA-2 diet on body weight, plasma lipid concentrations, and the frequency of sludge and gallstone formation were limited to male mice is unknown. As daily energy intake did not differ between the two diets, the energy expenditure and/or efficiency of digestion-absorption (digestibility) in male mice might be the source of the difference. It was reported [23] using naturally occurring CCK-1R gene deficient obese rats (OLETF rats) that castrated male OLETF rats showed lower body weight and castrated female OLETF rats showed higher body weight than non-treated animals of the respective sexes. Therefore, sex hormones such as testosterone may have an important role for body weight gain and/or obesity in rodents.

In Western countries, cholesterol gallstones are more common in women and are associated with multiparity and the use of contraceptive pills containing high doses of estrogen [6,24]. Moreover, a clinical report [25] showed that many laparoscopic cholecystectomies have been done in women. However, the genetic factors of gallstone disease vary by race, and the sex difference of gallstone disease is not so clear in Japan (male: female = 1:1.2) (5,26) as it is in Western countries. The reason why the male:female ratio was somewhat even in Japan is unknown.

In conclusion, male mice, regardless of presence or absence of CCK-1R, were more susceptible to diet-induced obesity and hyperlipidemia than female mice, and hyper-nutritional status induced and accelerated sludge and gallstone formation in male mice. Although the observation in mice may not apply to humans because rodents and humans have different eating habits (rodents nibble during the nighttime), the biological sex difference may be important to create metabolic syndrome,

## Competing interests

The author(s) declare that they have no competing interests.

## Authors' contributions

KM has made substantial contributions to conception and design, or acquisition of data, or analysis and interpretation of data; has involved in drafting the manuscript or revising it critically for important intellectual content; and has given final approval of the version to be published.

SK, AS, and SA carried out the animal experiments including determination of genotype. MO performed the statistical analysis. ST and AF conceived of the study, and participated in its design and coordination and helped to draft the manuscript. All authors read and approved the final manuscript.
